# Variation and potential overuse in thyroid ultrasound ordering: exploratory insights from a clinician survey

**DOI:** 10.1007/s12020-026-04742-0

**Published:** 2026-08-21

**Authors:** Sualeha Khalid, Alexandria Ratzki-Leewing, Juan P. Brito, Naykky Singh Ospina, Rozalina G. McCoy

**Affiliations:** 1 Division of Endocrinology, Diabetes, and Nutrition, Department of Medicine, University of Maryland School of Medicine, Baltimore, MD USA; 2 Division of Gerontology, Department of Epidemiology and Public Health, University of Maryland School of Medicine, Baltimore, MD USA; 3 University of Maryland Institute for Health Computing, North Bethesda, MD USA; 4 Division of Endocrinology, Diabetes, Metabolism, and Nutrition, Mayo Clinic, Rochester, MN USA; 5 Knowledge and Evaluation Research Unit, Division of Endocrinology, Diabetes, Metabolism, and Nutrition, Department of Medicine, Mayo Clinic, Rochester, MN USA; 6 Division of Endocrinology, Department of Medicine, University of Florida, Gainesville, FL USA

**Keywords:** Thyroid, Thyroid nodule, Thyroid ultrasound

## Abstract

**Purpose:**

Increasing use of thyroid ultrasound has contributed to the overdiagnosis of low-risk thyroid cancer which has in turn led to overtreatment as well. Despite established guidelines, deviations from evidence-based use persist. We evaluated clinician-reported practices, knowledge, and perceptions regarding thyroid ultrasound ordering.

**Methods:**

We conducted a pilot, exploratory, cross-sectional survey of clinicians at the University of Maryland Medical System. A 21-item electronic questionnaire assessed guideline familiarity, ordering practices, and perceived drivers of thyroid ultrasound use. The survey was distributed to internal medicine, family medicine, and endocrinology trainees, advanced practice providers, and faculty.

**Results:**

Thirty clinicians responded (43% internal medicine (IM), 40% endocrinology, 13% family medicine (FM), 3% IM and FM). One-third reported ordering 1–5 thyroid ultrasounds annually, while 20% reported ordering more than 50. Although 50% believed diagnostic testing improves outcomes, 47% perceived thyroid ultrasound as overused. Ordering was most driven by physical exam findings, compressive symptoms, and incidental nodules, rather than demographic factors or screening. Familiarity with American Thyroid Association guidelines exceeded that of American College of Radiology guidelines. Only 62% reported confidence in their own knowledge regarding thyroid ultrasound ordering.

**Conclusion:**

Clinicians recognize potential overuse of thyroid ultrasound, driven by multi-factorial influences. Gaps in familiarity and confidence may contribute to practice variation, highlighting opportunities for targeted interventions to promote high-value care.

**Supplementary Information:**

The online version contains supplementary material available at 10.1007/s12020-026-04742-0.

## Introduction

Thyroid ultrasound use has contributed to a rising detection and potential overtreatment of low-risk thyroid cancers [1]. Although thyroid nodules are common with a lifetime incidence of 30–60%, up to 46% of ultrasounds may be ordered outside guideline-based indications, contributing to low-value procedures, undue anxiety among patients, and increased healthcare costs [1–3]. While the clinical indications for thyroid ultrasound are well defined, the reasons leading clinicians to deviate from evidence-based guidance are poorly understood.

Prior studies identified common triggers for potentially inappropriate thyroid ultrasound use, including follow-up of incidental findings and patient symptoms, but deeper drivers including knowledge gaps, beliefs, external pressures, and systemic incentives are not well characterized [1, 2, 4]. To address this gap, we conducted an exploratory, cross-sectional survey to describe clinician-reported practices, perceptions, and knowledge related to thyroid ultrasound ordering, including perceived drivers of overuse and underuse and familiarity with existing guidelines.

## Methods

A team of clinicians and a survey methodologist developed a 21-question electronic questionnaire exploring participants’ knowledge about current guidelines and reasons for thyroid ultrasound orders. The questionnaire was programmed in Qualtrics and pretested by the research team for functionality prior to deployment in December 2024. Unique links to the questionnaire were emailed to trainees (residents and fellows), Advanced Practice Providers, and physician faculty clinicians in internal medicine (IM), family medicine (FM), and endocrinology at the University of Maryland Medical System (UMMS). Two reminders were sent to non-responders. The study was approved by the University of Maryland, Baltimore, Institutional Review Board. Survey completion implied consent; participation was voluntary and without compensation. Survey Questionnaire responses were de-identified prior to analysis.

## Results

Thirty clinicians responded to the survey: 13 (43%) IM, 12 (40%) endocrinology, 4 (13%) FM, 1 (3%) affiliated with IM and FM; Table 1. Ten respondents (33%) reported ordering 1–5 thyroid ultrasounds in the past year, while 8 (27%) and 6 (20%) had ordered 6–10 and > 50, respectively. While 15 clinicians (50%) agreed that diagnostic testing leads to better patient outcomes, 14 respondents (47%) felt that thyroid ultrasound is overused. Most clinicians did not think that patient age, sex or insurance status influenced their decision to order a thyroid ultrasound. Instead, most noted that they ordered an ultrasound based on physical exam findings, patient-reported compressive symptoms, and incidental nodules. Screening for thyroid nodules or cancer (in the absence of family history) was not regarded as an important driver of testing.

**Table 1:** Self-reported characteristics of survey respondents

Characteristic	*N* (%)
Role	
Attending physician Fellow physician IM residents FM residents Nurse practitioner	12 (40%) 3 (10%) 9 (30%) 2 (7%) 4 (13%)
Time in current role	
< 5 years 5–10 years 10–15 years 15–20 years 20 years or more	20 (67%) 1 (3% 4 (13%) 1 (3%) 4 (13%)
% Time in patient care	
0 < 20% 20 to < 40% 40 to < 60% 60 to < 80% 80 to < 100% 100%	2 (7%) 2 (7%) 1 (3%) 1 (3%) 13 (43%) 11 (37%)

**Fig. 1:**
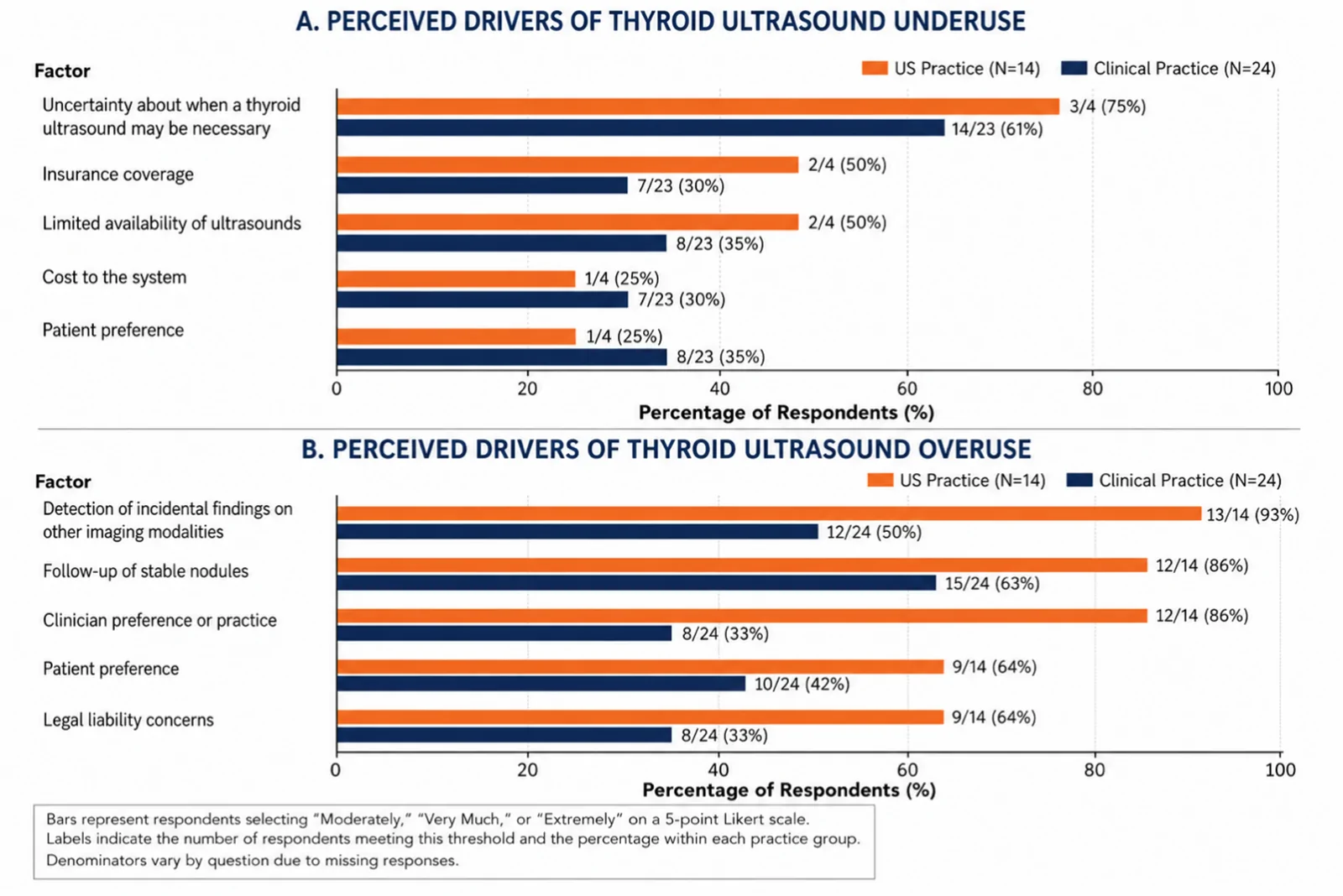
Factors cited by survey respondents as contributing to thyroid ultrasound underuse and overuse, both within their own practice and nationally in the U.S. Created using canva

Clinician-reported factors perceived as contributing to underuse and overuse of thyroid ultrasound, nationally and within their own practice, are presented in Fig. 1. More clinicians were familiar with American Thyroid Association (ATA) guidelines as compared to American College of Radiology (ACR). UpToDate was used as a resource “sometimes” to order thyroid ultrasound. While 76% noted that other clinicians in their practice were moderately to very knowledgeable regarding thyroid ultrasound ordering guidelines, just 62% described themselves as moderately to very confident in their own knowledge.

## Discussion

In this exploratory survey, nearly half of respondents, who were all front-line clinicians in IM, FM, or endocrinology, acknowledged overuse of thyroid ultrasound, underscoring its role in the overdiagnosis of low-risk thyroid cancer and its mitigation as a pathway to reduce overtreatment. Reported indications were varied-ranging from incidental findings and exam abnormalities to patient symptoms-with no single dominant driver, suggesting that overuse reflects overlapping factors rather than a single source. Broader influences such as legal concerns and patient expectations were also widely endorsed. Many clinicians reported gaps in guideline familiarity, likely reflecting both limited exposure and the relative ambiguity of existing recommendations.

This study has limitations, including a modest sample size, restriction to a single health system, and reliance on self-reported practices, which may introduce selection and reporting biases. One-third (33%) of the responding clinicians had only ordered a small number of thyroid ultrasounds in the prior year potentially limiting the breadth of their perspectives on the issue under investigation. The questionnaire was designed by the study team and pretested for clarity but not formally validated. Nonetheless, strengths include engagement of front-line clinicians across internal medicine, family medicine, and endocrinology, and attention to both practice patterns and perceptions. These findings provide formative insights into the clinical, cognitive, and contextual factors that may contribute to variation in thyroid ultrasound use and can inform future efforts to better understand and optimize practice.

## Supplementary information

Below is the link to the electronic supplementary material.

Supplementary Material 1

## References

[1] C. Soto Jacome, D. Segura Torres, J.W. Fan et al., Drivers of Thyroid Ultrasound Use: A Retrospective Observational Study. Endocr. Pract. **29**(12), 948–954 (2023). 10.1016/j.eprac.2023.09.006 37722595 10.1016/j.eprac.2023.09.006 PMC10843084

[2] N. Singh Ospina, S. Maraka, A.E. De Espinosa et al., Physical exam in asymptomatic people drivers the detection of thyroid nodules undergoing ultrasound guided fine needle aspiration biopsy. Endocrine 2016/11/01. **54**(2), 433–439 (2016). 10.1007/s12020-016-1054-y 10.1007/s12020-016-1054-y PMC5584598 27510173

[3] M.K. Edwards, N.M. Iñiguez-Ariza, N. Singh Ospina, E. Lincango-Naranjo, S. Maraka, J.P. Brito, Inappropriate use of thyroid ultrasound: a systematic review and meta-analysis. Endocrine Nov. **74**(2), 263–269 (2021). 10.1007/s12020-021-02820-z 10.1007/s12020-021-02820-z PMC10292117 34379311

[4] D.W. Chen, D. Reyes-Gastelum, A. Radhakrishnan, A.S. Hamilton, K.C. Ward, M.R. Haymart, Physician-Reported Misuse of Thyroid Ultrasonography. JAMA Surg. **155**(10), 984–986 (2020). 10.1001/jamasurg.2020.2507 32804996 10.1001/jamasurg.2020.2507 PMC7424547

