# Supplementary material for: Variation and potential overuse in thyroid ultrasound ordering: exploratory insights from a clinician survey
Source: Endocrine. 2026 Aug 21;91(1):283. doi: 10.1007/s12020-026-04742-0 (PMC13498611; doi:10.1007/s12020-026-04742-0)
Supplement: Supplementary file 1 [file 12020_2026_4742_MOESM1_ESM.docx]

CLINICIAN QUESTIONNARE

Title of Survey: Factors Driving Utilization and Inequities of Thyroid Ultrasound Use in Clinical Practice

**Q1.**

SINGLE RESPONSE

ASK ALL

What best describes your current healthcare provider role?

| **Code** | **Response Label** |
| --- | --- |
| 1 | Physician (Attending) |
| 2 | Resident  If yes, which residency are you in? (a) Internal Medicine (b) Family Medicine (c) Surgery (d) Other: ________ |
| 2 | Fellow  If yes, which fellowship are you in? (a) Endocrinology (b) Other: ________ |
| 4 | Physician Assistant |
| 5 | Nurse Practitioner |
| 6 | Other, please specify: ________________________ |

**Q2.**

SINGLE RESPONSE

ASK ALL

Approximately how long have you been a **[Pipe in Q1 response]**?

| **Code** | **Response Label** |
| --- | --- |
| 1 | <5 years |
| 2 | 5 to <10 years |
| 3 | 10 to <15 years |
| 4 | 15 to <20 years |
| 5 | 20 years or more |

**Q3.**

MULTI RESPONSE

ASK ALL

Are you currently affiliated with the University of Maryland School of Medicine (UMSOM) and/or the University of Maryland Medical System (UMMS)?

If yes, please select all the department(s) or clinical unit(s) with which you are affiliated.

Otherwise, select ‘*I am* ***not*** *currently affiliated with UMSOM or UMMS*’.

| **Code** | **Response Label** |
| --- | --- |
| 1 | Endocrinology |
| 2 | Internal Medicine or General Internal Medicine |
| 3 | Family Medicine |
| 6 | Other, please specify: ________________________ |
| 7 | I am **not** currently affiliated with UMSOM or UMMS  **[EXCLUSIVE]** |

**Q4.**

SINGLE RESPONSE

Do you currently see patients?

| **Code** | **Response Label** |
| --- | --- |
| 1 | Yes |
| 2 | No |

**Q5.**

SINGLE RESPONSE

**In an ordinary work week**, what percentage of your time do you spend on direct patient care?

| **Code** | **Response Label** |
| --- | --- |
| 1 | 0% |
| 2 | >0% to <20% |
| 3 | 20% to <40% |
| 4 | 40% to <60% |
| 5 | 60% to <80% |
| 6 | >80% to <100% |
| 7 | 100% |

**Q6.**

SINGLE RESPONSE

In your clinical practice, to what extent do you agree or disagree with the following statement:

**In general**, diagnostic testing (such as x-rays, ultrasounds, CT scans, etc) leads to better patient outcomes.

| **Code** | **Response Label** |
| --- | --- |
| 1 | Strongly agree |
| 2 | Somewhat agree |
| 3 | Neither agree nor disagree |
| 4 | Somewhat disagree |
| 5 | Strongly disagree |

**Q7.**

SINGLE RESPONSE

**In the past year**, approximately how many thyroid ultrasounds did you order?

| **Code** | **Response Label** |
| --- | --- |
| 1 | 0 |
| 2 | 1 to 5 |
| 3 | 6 to 10 |
| 4 | 11 to 15 |
| 5 | 16 to 20 |
| 6 | 21 to 30 |
| 7 | 31 to 50 |
| 8 | More than 50 |

**Q8.**

SINGLE RESPONSE

ASK ALL

There are multiple reasons why a clinician might order a thyroid ultrasound.

Many of these reasons are listed below.

How important do you think each of these reasons is when determining whether to order a thyroid ultrasound?

| **Code** | **Response Label** |  |  |  |  |  |
| --- | --- | --- | --- | --- | --- | --- |
|  | RANDOMIZE | Not important at all | Slightly important | Moderately important | Very important | Essential |
| 1 | Evaluation of physical examination findings | 1 | 2 | 3 | 4 | 5 |
| 2 | Evaluation of patient-reported compression symptoms (for example: hoarseness, dysphagia) | 1 | 2 | 3 | 4 | 5 |
| 3 | Work up of hypothyroidism in patients *without* thyroid antibodies | 1 | 2 | 3 | 4 | 5 |
| 4 | Work up of hypothyroidism in patients *with* thyroid antibodies | 1 | 2 | 3 | 4 | 5 |
| 5 | Work up of hyperthyroidism | 1 | 2 | 3 | 4 | 5 |
| 6 | Work up of nodules incidentally found in non-thyroid imaging modalities (for example: chest computer tomography [CT] ordered for evaluation of lung concerns) | 1 | 2 | 3 | 4 | 5 |
| 7 | Thyroid nodule or thyroid cancer screening in asymptomatic patients with family history of thyroid disease (non-cancerous) | 1 | 2 | 3 | 4 | 5 |
| 8 | Thyroid nodule or thyroid cancer screening in asymptomatic patients with family history of thyroid cancer | 1 | 2 | 3 | 4 | 5 |
| 9 | Thyroid nodule or thyroid cancer screening in asymptomatic individuals without family history | 1 | 2 | 3 | 4 | 5 |
| 10 | Patient request for thyroid nodule or thyroid cancer screening | 1 | 2 | 3 | 4 | 5 |

**Q9.**

SINGLE RESPONSE

ASK ALL

Often the decision to order thyroid ultrasound is influenced by patient specific factors.

All other factors being equal, are you more or less likely to order a thyroid ultrasound in a patient who is:

| **Code** | **Response Label** |  |  |  |  |  |
| --- | --- | --- | --- | --- | --- | --- |
|  |  | Much less likely | Somewhat less likely | Neutral | More Likely | Much more Likely |
| 1 | Age < 40 years | 1 | 2 | 3 | 4 | 5 |
| 2 | Age > 60 years | 1 | 2 | 3 | 4 | 5 |
| 3 | Male | 1 | 2 | 3 | 4 | 5 |
| 4 | Female | 1 | 2 | 3 | 4 | 5 |
| 5 | Insured | 1 | 2 | 3 | 4 | 5 |
| 6 | Uninsured | 1 | 2 | 3 | 4 | 5 |

**Q10.**

SINGLE RESPONSE

ASK ALL

**Thinking about healthcare in general in the United States**, do you think thyroid ultrasound as a diagnostic test to evaluate potential thyroid nodules is underused, appropriately used, or overused?

| **Code** | **Response Label** |
| --- | --- |
| 1 | Underused |
| 2 | Appropriately used |
| 3 | Overused |
| 4 | I don’t have an opinion |

**Q11.**

SINGLE RESPONSE

ASK IF SELECTED ‘Underused’ AT Q10

To what extent do you think the following factors contribute to the **underuse** of thyroid ultrasound (i.e., not ordering enough ultrasounds and potentially missing clinically important cases of thyroid nodules or thyroid cancer, or diagnosing cancer too late) in United States?

| **Code** | **Response Label** |  |  |  |  |  |
| --- | --- | --- | --- | --- | --- | --- |
|  | RANDOMIZE | Not at all | Slightly | Moderately | Very much | Extremely |
| 1 | Limited availability of ultrasounds | 1 | 2 | 3 | 4 | 5 |
| 2 | Patient preference | 1 | 2 | 3 | 4 | 5 |
| 3 | Insurance coverage | 1 | 2 | 3 | 4 | 5 |
| 4 | Cost to the system | 1 | 2 | 3 | 4 | 5 |
| 5 | Clinician knowledge regarding indications for thyroid ultrasound |  |  |  |  |  |
| 6 | Other, please specify: ____________________ | 1 | 2 | 3 | 4 | 5 |

**Q12.**

SINGLE RESPONSE

ASK IF SELECTED ‘Overused’ AT Q10

To what extent do you think the following factors contribute to the **overuse** of thyroid ultrasound in the United States?

| **Code** | **Response Label** |  |  |  |  |  |
| --- | --- | --- | --- | --- | --- | --- |
|  | RANDOMIZE | Not at all | Slightly | Moderately | Very much | Extremely |
| 1 | Detection of incidental findings on other imaging modalities | 1 | 2 | 3 | 4 | 5 |
| 2 | Patient preference | 1 | 2 | 3 | 4 | 5 |
| 4 | Clinician preference or practice |  |  |  |  |  |
| 3 | Continued follow up of stable thyroid nodules | 1 | 2 | 3 | 4 | 5 |
| 4 | Legal liability concerns | 1 | 2 | 3 | 4 | 5 |
| 5 | Other, please specify:  ____________________ | 1 | 2 | 3 | 4 | 5 |

**Q13.**

SINGLE RESPONSE

ASK ALL

Now, **thinking specifically about your own practice**, how often, if ever, are you concerned about **underusing** thyroid ultrasound as a diagnostic test?

| **Code** | **Response Label** |
| --- | --- |
| 1 | Always |
| 2 | Often |
| 3 | Sometimes |
| 4 | Rarely |
| 5 | Never |

**Q14.**

SINGLE RESPONSE

ASK IF SELECTED ‘Always’, ‘Often’, ‘Sometimes’, ‘Rarely’ AT Q13

To what extent do the following factors contribute to your own concerns about **underusing** thyroid ultrasound in your practice?

| **Code** | **Response Label** |  |  |  |  |  |
| --- | --- | --- | --- | --- | --- | --- |
|  | RANDOMIZE | Not at all | Slightly | Moderately | Very much | Extremely |
| 1 | Limited availability of ultrasounds | 1 | 2 | 3 | 4 | 5 |
| 2 | Patient preference | 1 | 2 | 3 | 4 | 5 |
| 3 | Insurance coverage | 1 | 2 | 3 | 4 | 5 |
| 4 | Cost to the system | 1 | 2 | 3 | 4 | 5 |
| 5 | Uncertainty about when a thyroid ultrasound may be necessary |  |  |  |  |  |
| 6 | Other, please specify: ____________________ | 1 | 2 | 3 | 4 | 5 |

**Q15.**

SINGLE RESPONSE

ASK ALL

**Continuing to think about your own practice**, how often, if ever, are you concerned about **overusing** thyroid ultrasound as an initial diagnostic test?

| **Code** | **Response Label** |
| --- | --- |
| 1 | Always |
| 2 | Often |
| 3 | Sometimes |
| 4 | Rarely |
| 5 | Never |

**Q16.**

SINGLE RESPONSE

ASK IF SELECTED ‘Always’, ‘Often’, ‘Sometimes’, ‘Rarely’ AT Q15

To what extent do the following factors contribute to your own concerns about **overusing** thyroid ultrasound in your practice?

| **Code** | **Response Label** |  |  |  |  |  |
| --- | --- | --- | --- | --- | --- | --- |
|  | RANDOMIZE | Not at all | Slightly | Moderately | Very much | Extremely |
| 1 | Detection of incidental findings on other imaging modalities | 1 | 2 | 3 | 4 | 5 |
| 2 | Patient preference | 1 | 2 | 3 | 4 | 5 |
| 4 | Clinician preference or practice |  |  |  |  |  |
| 3 | Continued follow up of stable thyroid nodules | 1 | 2 | 3 | 4 | 5 |
| 4 | Legal liability concerns | 1 | 2 | 3 | 4 | 5 |
| 5 | Other, please specify:  ____________________ | 1 | 2 | 3 | 4 | 5 |

**Q17.**

SINGLE RESPONSE

ASK ALL

The next set of questions aims to evaluate the accessibility of provider-based resources for thyroid ultrasound in the United States.

How familiar are you with each of the following clinical practice guidelines on thyroid care?

| **Code** | **Response Label** |  |  |  |  |  |
| --- | --- | --- | --- | --- | --- | --- |
|  | RANDOMIZE | Never | Rarely | Sometimes | Often | Always |
| 1 | American Association of Clinical Endocrinology (AACE) guidelines | 1 | 2 | 3 | 4 | 5 |
| 2 | American College of Physicians (ACP) Guidelines | 1 | 2 | 3 | 4 | 5 |
| 3 | American College of Radiology (ACR) Guidelines | 1 | 2 | 3 | 4 | 5 |
| 4 | American Thyroid Association (ATA) guidelines | 1 | 2 | 3 | 4 | 5 |
| 5 | Endocrine Society Guidelines | 1 | 2 | 3 | 4 | 5 |
| 6 | National Comprehensive Cancer Network (NCCN) Guidelines | 1 | 2 | 3 | 4 | 5 |
| 7 | Other, please specify: |  |  |  |  |  |

**Q18.**

SINGLE RESPONSE

How frequently do you use the resources below when ordering a thyroid ultrasound?

ASK ALL

| **Code** | **Response Label** |  |  |  |  |  |
| --- | --- | --- | --- | --- | --- | --- |
|  | RANDOMIZE | Never | Rarely | Sometimes | Often | Always |
| 1 | UpToDate | 1 | 2 | 3 | 4 | 5 |
| 2 | Institutional guidance or resource | 1 | 2 | 3 | 4 | 5 |
| 3 | Electronic health record prompts | 1 | 2 | 3 | 4 | 5 |
| 4 | Other, please specify: |  |  |  |  |  |

**Q19.**

SINGLE RESPONSE

ASK ALL

How would you rate the degree of knowledge regarding thyroid ultrasound ordering guidelines among clinicians in your practice?

| **Code** | **Response Label** |
| --- | --- |
| 1 | Extremely adequate |
| 2 | Very adequate |
| 3 | Moderately adequate |
| 4 | Slightly adequate |
| 5 | Not adequate at all |

**Q20.**

SINGLE RESPONSE

ASK ALL

How would you rate YOUR degree of knowledge regarding thyroid ultrasound ordering guidelines?

| 2 | Very confident |
| --- | --- |
| 3 | Moderately confident |
| 4 | Slightly confident |
| 5 | Not confident at all |

Thank you for completing this survey.

**---END---**
